# Realizing 303 ps Ultrafast Scintillation Time in 2-Inch CsPbCl_3_ Single Crystals Grown Under Br_2_ Overpressure

**DOI:** 10.3390/ma19081479

**Published:** 2026-04-08

**Authors:** Jingwei Yang, Fangbao Wang, Liang Chen, Tao Bo, Zhifang Chai, Wenwen Lin

**Affiliations:** 1 Zhejiang Key Laboratory of Data-Driven High-Safety Energy Materials and Applications, Ningbo Key Laboratory of Special Energy Materials and Chemistry, Laboratory of Advanced Nuclear Materials, Ningbo Institute of Materials Technology and Engineering (NIMTE), Chinese Academy of Sciences, Ningbo 315201, China; yangjingwei@nimte.ac.cn (J.Y.); botao@nimte.ac.cn (T.B.); chaizhifang@nimte.ac.cn (Z.C.); 2 University of Chinese Academy of Sciences, Beijing 100049, China; 3 Qianwan Institute, Ningbo Institute of Materials Technology and Engineering (NIMTE), Ningbo 315336, China; 4 State Key Laboratory of Intense Pulsed Radiation Simulation and Effect and Radiation Detection Research Center, Northwest Institute of Nuclear Technology, Xi’an 710024, China; wfb0619@163.com (F.W.); chenl_nint@163.com (L.C.)

**Keywords:** CsPbCl_3_ single crystal, photoluminescence, ultrafast scintillator

## Abstract

Large-sized, room-temperature ultrafast scintillator single crystals are highly demanded for fast timing applications such as time of flight–positron emission tomography, high-speed medical imaging, and pulse heavy-ray detection. Sub-nanosecond scintillation was discovered in 16 mm sized CsPbCl_3_Br_x_ single crystals in our previous research. In this work, the crystal size of CsPbCl_3_Br_0.03_ was enlarged to 2 inches (50.8 mm). Meanwhile, by precisely optimizing the vertical Bridgman growth process, we further increased the concentration of Br dopant to realize even faster scintillation decay. In this study, we conducted a series of tests on the grown crystals, including temperature-dependent photoluminescence tests, alpha particle excitation tests, X-ray imaging tests, etc. Via the strategy of the incorporation of Br_2_, Br dopant introduces highly efficient fast recombination centers in perovskite CsPbCl_3_Br_0.03_ crystals, resulting in an unprecedently fast scintillation decay time of 303 ps under ^241^Am *α*-particle excitation, which is significantly shorter than that of the pure CsPbCl_3_ and all other perovskites by at least two orders of magnitude. Benefiting from the excellent optical transparency and high crystalline quality of the CsPbCl_3_Br_0.03_ crystal, an X-ray spatial resolution of up to 20 lp/mm is achieved. These results further demonstrate the great potential of large-sized CsPbCl_3_Br_x_ single crystals for fast timing applications.

## 1. Introduction

Large-sized, high-quality, and ultrafast scintillation crystals serve as the core sensing materials in radiation detection and play an indispensable role in fast timing fields such as medical imaging (e.g., time of flight–positron emission tomography, TOF-PET [1,2,3]), high-speed X-ray imaging, and pulse neutron detection [4,5,6,7]. Consequently, there is growing interest in the development of novel scintillator materials exhibiting sub-nanosecond decay times. For example, Ouyang and co-workers successfully grew ZnO:Ga crystals via a hydrothermal method, achieving an ultrafast scintillation decay time of 600 ps under *α*-particle irradiation [8]. Mykhaylyk discovered that, at low temperatures, CsPbBr_3_ is a very bright scintillator and has a rapid decay time of 1 ns, however, this fast decay occurs at a low temperature of 7 K [9].

Taking advantage of the high defect tolerance and low-cost growth of the perovskite semiconductor CsPbCl_3_ [10,11,12,13,14,15], our group previously proposed a “Br overdoping” strategy and successfully achieved sub-nanosecond ultrafast scintillation at room temperature on 16 mm sized CsPbCl_3_Br_x_ single crystals [16]. However, the diameter of CsPbCl_3_Br_0.03_ single crystals was only 16 mm. This small crystal size represents a significant drawback, as fast timing applications urgently require larger and low-cost crystals [17,18,19,20]. And the concentration of Br incorporated into the crystal lattice of our group’s earlier research remained relatively low. So, we further increase the concentration of Br incorporated into the crystal lattice, aiming to achieve even faster scintillation decay.

Motivated by these considerations, our present study focuses on the growth of large-sized CsPbCl_3_Br_x_ single crystals with a diameter of 2 inches, while simultaneously realizing a higher concentration of Br incorporated into the crystal lattice than previous research. However, growing crystals with a large size inevitably introduces technical challenges [21,22,23,24]. To overcome the challenges, our research optimizes the crystal growth conditions. Specifically, compared with our previous research, we employ a smaller temperature gradient and a slower cooling speed in the crystallization process. Furthermore, in this study, we rotated the quartz ampoule during the crystal growth process to improve the crystalline homogeneity. By these improvements, we successfully obtained highly transparent 2-inch CsPbCl_3_Br_0.03_ single crystals. As the concentration of Br incorporated into the crystal lattice is further increased, the decay time of the CsPbCl_3_Br_x_ single crystal is significantly shortened. It was measured through *α*-particle excitation: the decay time of the CsPbCl_3_Br_x_ crystal has been significantly reduced from the 630 ps in our group’s previous research to 303 ps in this research, which is significantly shorter than that of the pure CsPbCl_3_ and all other perovskites by at least two orders of magnitude [13]. In this study, we not only successfully prepared the CsPbCl_3_Br_0.03_ single crystal with a diameter of 2 inches but also conducted a systematic characterization and investigated the mechanism of ultrafast scintillation of this material. The research content covers various aspects such as the lattice expansion, carrier transport, photoluminescence properties, light yield, and X-ray imaging performance. The research results indicate that CsPbCl_3_Br_0.03_ single crystals possess significant potential in the field of fast timing and large-sized imaging applications.

## 2. Materials and Methods

### 2.1. Material Preparation

The chemical reagents used in this research include (1) cesium chloride 99.999%, Aladdin; (2) lead chloride 99.999%, Aladdin; (3) cesium tribromide (synthesized by the chemical reaction of liquid bromine and cesium bromide). The details of the synthesis are elaborated in the SI.

### 2.2. Crystal Growth

**Figure 1:**
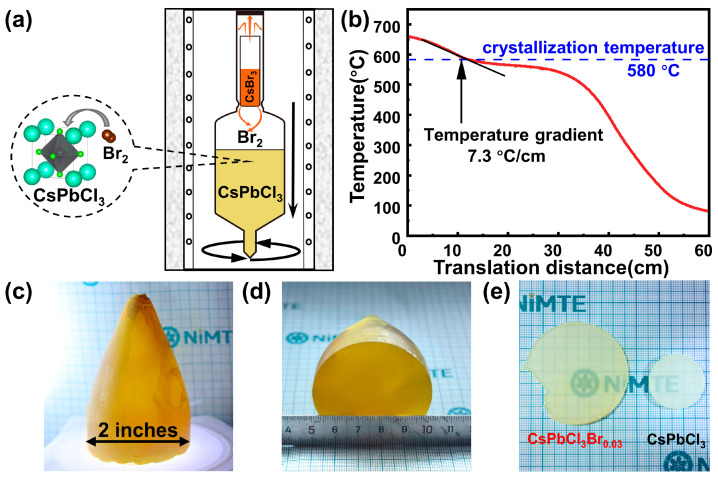
(**a**) Schematic diagram of the vertical Bridgman growth process for single crystals of CsPbCl_3_Br_0.03_ grown under Br_2_ overpressure. The arrows indicate the downward movement of the ampoule and the rotation of the ampoule during growth. (**b**) Temperature–position curve of CsPbCl_3_Br_0.03_ crystal growth. (**c**,**d**) The as-grown 2-inch CsPbCl_3_Br_0.03_ single crystal ingots. (**e**) Comparison photos of wafer samples of CsPbCl_3_Br_0.03_ and CsPbCl_3_ with the same thickness of 2.0 mm.

Figure 1a presents a schematic diagram of the vertical Bridgman growth process for CsPbCl_3_ single crystals grown under Br_2_ overpressure. We precisely weighed the masses of CsCl and PbCl_2_ in a glovebox filled with inert gas, in a 1:1 molar ratio, and placed them into a quartz ampoule with an inner diameter of 21 mm and an outer diameter of 25 mm. Subsequently, the ampoule was placed in a tube furnace and reacted at a temperature of 700 °C to synthesize homogeneous polycrystalline CsPbCl_3_. According to the stoichiometric ratio of CsPbCl_3_Br_0.03_, the precisely weighed polycrystalline precursor of CsPbCl_3_ was placed into a customized quartz ampoule with an inner diameter of 2 inches and a conical tip. Meanwhile, CsBr_3_ was placed in a small crucible above the raw material, as shown in Figure 1a. When the temperature rises, CsBr_3_ in the crucible decomposes into CsBr and Br_2_ vapor. The white powder of the decomposed CsBr still remains in the upper crucible; the Br_2_ vapor released diffuses in the ampoule and enters the lattice of the CsPbCl_3_ crystal during its growth process as a dopant.

During the growth process of large-sized crystals, since the crystal size is 2 inches, the thermal conduction of solidification heat would be more difficult. This will result in insufficient cooling at the interface or thermal stress cracking. Therefore, for the growth of large-sized CsPbCl_3_Br_0.03_ single crystals, a lower temperature gradient and a slower decreasing rate are required [21,22,23,24]. We heated the three-zone Bridgman furnace at a rate of 2 °C/min, heating each of the three temperature zones of the furnace to the preset temperatures of 670 °C, 570 °C, and 500 °C, respectively. A temperature gradient of 7.6 °C/cm was established near the crystallization region. According to our group’s previous research, the crystallization point of the CsPbCl_3_Br_x_ single crystals fluctuates between 583 °C and 596 °C [16]. In this research, due to the higher concentration of Br incorporated into the crystal lattice, we precisely set a lower descent rate (0.40 mm/h) starting from 580 °C, as shown in Figure 1b.

The crystal growth process mainly consists of the following steps: firstly, the ampoule is slowly lowered at a rate of 5.0 mm/h for 22 h to ensure that the raw materials are completely melted and the temperature field is stabilized; secondly, at a temperature close to the crystallization point, the crystallization reaction is carried out at a rate of 0.40 mm/h for 350 h; finally, after full crystallization, the crystal is cooled to room temperature at a rate of 2.0 mm/h. Pure and colorless CsPbCl_3_ crystals are grown under the same growth conditions, as shown in Figure S1. After the crystallization process was completed, there was no remaining red-brown Br_2_ vapor in the ampoule. This indicates that all the Br_2_ was fully incorporated into the crystal structure. Based on this complete consumption of Br_2_, the CsPbCl_3_ single crystals grown under Br_2_ overpressure have a nominal composition of CsPbCl_3_Br_0.03_. The as-grown 2-inch CsPbCl_3_Br_0.03_ ingot is shown in Figure 1c,d, presenting a light-yellow color due to the incorporation of Br dopant. The ingots are further processed into uniformly thick wafers using a diamond wire saw, as shown in Figure 1e. The single crystals grown under Br_2_ overpressure still exhibit excellent optical transparency.

## 3. Results and Discussion

### 3.1. Structural and Optical Characterization

**Figure 2:**
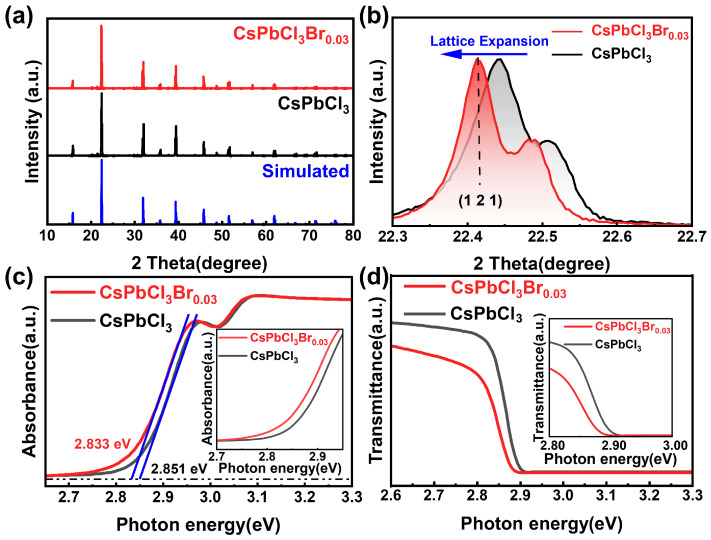
(**a**,**b**) Powder X-ray diffraction (PXRD) patterns of CsPbCl_3_ and CsPbCl_3_Br_0.03_ crystals. (**c**) UV-Vis optical absorption spectra of CsPbCl_3_ and CsPbCl_3_Br_0.03_ crystals. The blue lines represent the tangent lines applied to the linear region for bandgap determination. (**d**) UV-Vis optical transmittance spectra of CsPbCl_3_ and CsPbCl_3_Br_0.03_ crystals.

To verify the uniformity of Br distribution across the 2-inch ingot, we performed SEM-EDS elemental mapping on samples taken both from various axial and radial positions. The results demonstrate a highly consistent and uniform distribution of Cs, Pb, Cl, and Br elements throughout the crystal bulk, as shown in Figure S6. The phase compositions of the crystals before and after growth under Br_2_ overpressure are analyzed using the powder X-ray diffraction (PXRD) technique. As shown in Figure 2a, all the diffraction peaks of the CsPbCl_3_Br_0.03_ are in perfect agreement with those of the pure phase CsPbCl_3_ and the simulated standard diffraction pattern of the room-temperature CsPbCl_3_ phase. We also conducted PXRD analyses on specimens extracted from different sections of the ingot. These results revealed highly consistent diffraction patterns without peak shift, confirming that the distribution of Br element was uniform throughout the ingot. The PXRD results from other positions are shown in Figure S4. With the incorporation of Br, the diffraction peaks slightly shift to lower angles, such as the position of the (1 2 1) crystal plane diffraction peak, which shifts from 22.445° to a smaller angle of 22.413°, indicating a slight expansion of the lattice, as shown in Figure 2b. The change in bandgap is estimated by comparing the ultraviolet–visible (UV-Vis) optical absorption spectra. To ensure the accuracy of this determination of the bandgap and avoid common misconceptions related to the Urbach tail or defect-level absorption, the tangent lines were applied to the steep linear region corresponding to fundamental band-to-band transitions [25]. The bandgap of CsPbCl_3_Br_0.03_ decreased from 2.851 eV in the pure phase CsPbCl_3_ to 2.833 eV, as shown in Figure 2c. The UV-Vis optical transmittance spectra also reflected the reduction in bandgap after the incorporation of Br_2_ (Figure 2d). The reduction in bandgap caused by the incorporation of Br_2_ is due to the expansion of the crystal lattice. The expansion of the lattice increases the distance between atoms, weakens the interaction between atoms, and reduces the overlap of electron wave functions, thereby lowering the energy required for electrons to transition from the valence band to the conduction band and consequently reducing the bandgap of the semiconductor. Therefore, generally speaking, as the lattice expands, the bandgap decreases [26,27,28].

### 3.2. Charge Transport Property

**Figure 3:**
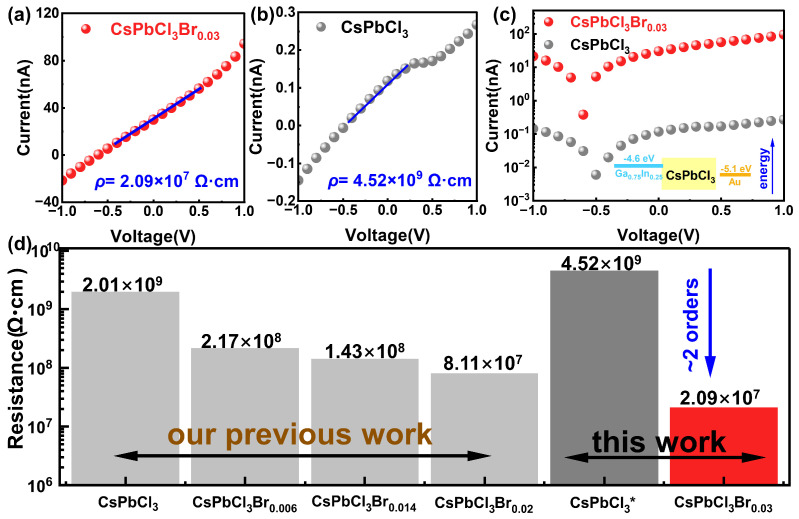
(**a**,**b**) The dark current-voltage (*I-V*) characteristics of CsPbCl_3_Br_0.03_ and CsPbCl_3_ crystals, respectively. (**c**) Comparison of the dark *I-V* characteristics of the crystals before and after the incorporation of Br_2_. (**d**) Comparison of the resistivity of the crystals grown in this work with that of similar crystals reported in our group’s previous work. The CsPbCl_3_* denotes the pure CsPbCl_3_ sample synthesized in this work.

To evaluate the charge transport properties of the CsPbCl_3_ and CsPbCl_3_Br_0.03_ crystals, current-voltage (*I-V*) characteristics are measured under dark conditions, as shown in Figure 3. An asymmetric electrode structure is employed on both sides of the crystal. Specifically, on the two surfaces of the polished wafer, a thin layer of Au is deposited using magnetron sputtering on one side, while a liquid Ga_0.75_In_0.25_ alloy is applied on the other side. The work function difference between these two metal electrodes is approximately 0.5 eV. However, since both resistivity of CsPbCl_3_ and CsPbCl_3_Br_0.03_ are not high enough, a high-quality Schottky junction has not been fabricated [13].

The test results show that the resistivity of the CsPbCl_3_Br_0.03_ single crystal with a thickness of 0.98 mm and an electrode area of 10.5 mm^2^ is 2.09 × 10^7^ Ω·cm. Compared with the CsPbCl_3_ (4.52 × 10^9^ Ω·cm) with the same thickness and electrode area, its resistivity has decreased by two orders of magnitude. This further reduction in resistivity when compared with similar samples in our group’s previous work indicates that a higher concentration of Br incorporated into the crystal lattice has been achieved in this work [16]. In addition, *I-V* characteristics were performed at different positions of the crystal, and the results confirmed the uniform incorporation of Br within the crystal, as shown in Figure S7. Considering the theoretical calculations (as shown in Figure S5) [29,30,31,32,33], we believe the significant reduction in resistivity may be due to the excessive Br_2_ molecules occupying the interstitial sites in the lattice. Due to their strong electronegativity, these Br_2_ act as effective acceptor centers, capturing electrons from the valence band and introducing a high concentration of holes. This hole concentration far exceeds the intrinsic carrier concentration in the pure state, resulting in a significant increase in the background carrier density [34].

### 3.3. Steady-State and Time-Resolved Transient Photoluminescence Spectra

**Figure 4:**
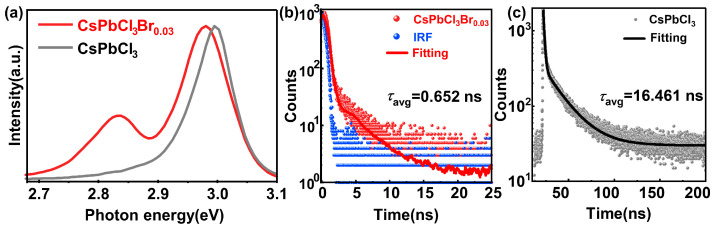
(**a**) Photoluminescence spectra of CsPbCl_3_ and CsPbCl_3_Br_0.03_ crystals. (**b**) Time-resolved transient photoluminescence decay time of the CsPbCl_3_Br_0.03_ crystal under 375 nm laser excitation (power: 5 mW). (**c**) Time-resolved transient photoluminescence decay time of the CsPbCl_3_ crystal under 375 nm laser excitation (power: 5 mW).

Steady-state and time-resolved transient photoluminescence (PL) spectroscopy techniques are crucial methods for revealing the radiative recombination mechanisms and carrier dynamics in luminescent materials. By using a 375 nm laser as the excitation source, we discovered that incorporation of Br_2_ would cause significant changes in the emission spectra and decay time of the CsPbCl_3_ crystals. As shown in Figure 4a, the pure CsPbCl_3_ crystal has a single emission peak at a position of 2.995 eV. The CsPbCl_3_Br_0.03_ crystal exhibits a double peak emission, with the two peaks located at 2.981 eV and 2.831 eV. Based on the test results, we observed that, after the incorporation of Br_2_, the main emission peak shifted slightly from the original 2.995 eV to 2.981 eV. This is due to the slight lattice expansion caused by excessive Br_2_. It is worth noting that a distinct, lower-energy emission peak appeared in the CsPbCl_3_Br_0.03_ crystal, with the emission peak energy approximately at 2.831 eV. However, this peak was absent in the pure CsPbCl_3_ crystal. This change in the emission spectrum indicates that the incorporation of Br has created a different luminescent environment, thereby causing alterations in the crystal structure and radiation energy levels.

Under the excitation light of 375 nm, the time-resolved transient photoluminescence decay times of the crystals before and after incorporation of Br_2_ are shown in Figure 4b and Figure 4c respectively. Compared with the CsPbCl_3_ crystal, the decay time of the CsPbCl_3_Br_0.03_ crystal is significantly shortened. The decay curves are fitted to obtain the photoluminescence decay time [35]. The average photoluminescence decay time of CsPbCl_3_Br_0.03_ decreased sharply from the 16.461 ns of CsPbCl_3_ to 0.652 ns, which is equivalent to a reduction of nearly one order of magnitude. The significant shortening of the decay time is consistent with the fast emission process caused by the bound excitons. This indicates that the Br_2_ molecules with strong electronegativity have introduced shallow trap centers that can serve as efficient recombination sites, facilitating rapid and efficient radiative recombination processes [36].

Based on the theoretical calculations mentioned earlier, we think that the strong electronegative interstitial Br_2_ molecules in CsPbCl_3_Br_0.03_ bring about two significant impacts. Electrically, they function as effective acceptor centers, capable of capturing electrons from the valence band, thereby generating a large number of holes and significantly reducing the resistivity of the crystal. From a kinetic perspective, this hole-rich environment creates efficient shallow-level defects, which serve as efficient recombination pathways to achieve fast decay. This explanation provides a unified interpretation for both electrical and optical measurements. The sub-nanosecond photoluminescence decay observed in the CsPbCl_3_Br_0.03_ crystal highlights its strong potential for fast timing applications.

### 3.4. Power-Dependent Photoluminescence

**Figure 5:**
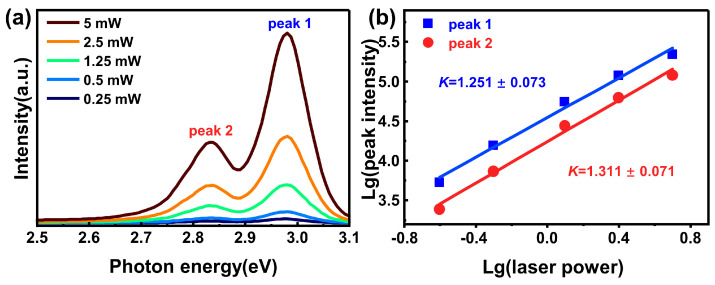
(**a**) Room-temperature steady-state photoluminescence spectra of the CsPbCl_3_Br_0.03_ crystal under 375 nm laser excitation at different powers. (**b**) Graph showing the variation of *lg* (peak intensity) in CsPbCl_3_Br_0.03_ crystal with *lg* (laser power), along with the corresponding linear fitting curve.

To gain a deeper understanding of the underlying mechanism of faster scintillation decay and radiation transition evolution caused by excessive Br, power-dependent photoluminescence measurements were performed at room temperature. As shown in Figure 5a, as the excitation power increased, the intensity of the photoluminescence peak significantly increased, indicating a direct correlation between excitation power and emission intensity. In general, the emission intensity (*I*) follows a power-law dependence on the excitation power (*L*), expressed as *I∝L^k^*, where the exponent *k* can be used to identify the physical origin of radiative recombination. In the case of *0* < *k* < *1*, the emission band can be ascribed to donor–acceptor pair recombination (DAP) or free-bound radiative recombination. When *1* < *k* < *2*, the emission stems from transitions of free excitons or bound excitons [37,38].

We analyze the power-dependent photoluminescence spectra by using the Gaussian multi-peak fitting method. The results show that CsPbCl_3_Br_0.03_ crystal has two overlapping emission peaks. The higher energy peak is labeled as peak 1, while the lower energy peak is labeled as peak 2. As shown in the fitting results in Figure 5b, the values of *k* fitted for the two emission peaks of the CsPbCl_3_Br_0.03_ crystal are 1.251 and 1.311. Both values were within the range of *1* < *k* < *2*, which confirmed that these emissions originated from the exciton recombination process. Based on the energy level structure and kinetic energy characteristics, the bound excitons typically have lower energy states and exhibit a faster decay time compared to those in the free state. Therefore, the high-energy emission peak 1 is attributed to the recombination of free excitons, while the low-energy emission peak 2 is attributed to the emission of bound excitons. This result provides a unified explanation for the observed spectral changes: in the CsPbCl_3_Br_0.03_ crystal, the bound excitation peak 2 at 2.831 eV indicates that the incorporation of Br_2_ effectively introduces shallow-level defects, thereby significantly increasing the probability of excitons being captured and undergoing radiative recombination through the faster bound state.

### 3.5. Temperature-Dependent Photoluminescence

**Figure 6:**
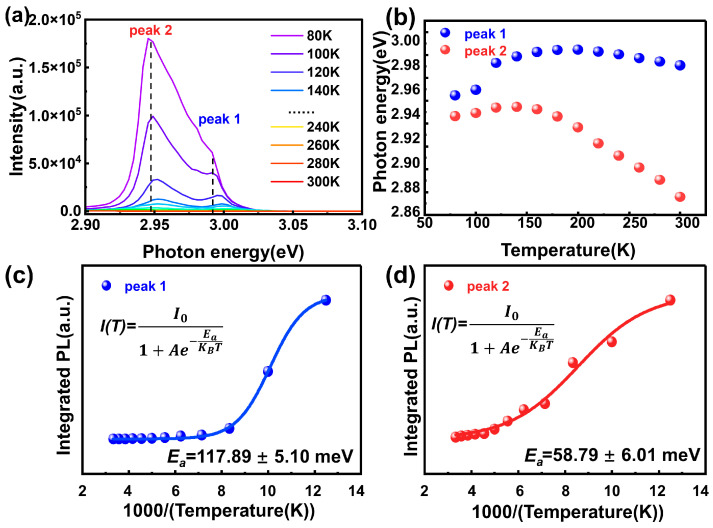
(**a**) Temperature-dependent photoluminescence spectra of the CsPbCl_3_Br_0.03_ crystal under 375 nm laser excitation. (**b**) The position of the two emission peaks as a function of temperature obtained by Gaussian decomposition. (**c**,**d**) Arrhenius plots and fitting curves of the integrated PL intensities for peak 1 and peak 2, respectively, where *E_a_* represents the extracted thermal activation energy.

To further investigate the exciton dynamic characteristics in the single crystals grown under Br_2_ overpressure, we conducted temperature-dependent photoluminescence measurements within the temperature range of 80 to 300 K (with a step size of 20 K), as shown in Figure 6a. Due to the existence of phonon-assisted non-radiative relaxation processes, the overall photoluminescence intensity gradually decreases as the temperature rises [39].

By performing Gaussian multi-peak fitting on the temperature-dependent photoluminescence spectra, two overlapping emission peaks were obtained. This result is consistent with the previous analysis results of the power-dependent photoluminescence. The peak with higher energy is labeled as peak 1, while the peak with lower energy is labeled as peak 2, as shown in Figure 6b. We have meticulously recorded the changes in the integrated intensity and peak positions of these two peaks with temperature.

To gain a deeper understanding of the influence of Br incorporated into the crystal lattice on the luminescence mechanism of CsPbCl_3_ single crystals, we used the Arrhenius equation to fit the relationship between the integral PL intensity of the two exciton peaks and the temperature:

(1) $$I(T)=\frac{I_{0}}{1+Ae^{-E_{a}/K_{B}T}}$$

Here, *I*(*T*) represents the integrated intensity of the photoluminescence peak at temperature *T*, and *I*_0_ represents the integrated peak intensity at the reference temperature. *E_a_* is the thermal activation energy that needs to be determined, and *K_B_* is the Boltzmann constant (8.6 × 10^−5^ eV·K^−1^) [37,40,41].

The fitting results show that the Br incorporated into the crystal lattice has produced an emission peak with an exceptionally high thermal activation energy, a phenomenon that has not been reported before. For the higher energy emission peak 1, the thermal activation energy obtained from Arrhenius fitting is 117.89 eV, as shown in Figure 6c. This value is significantly higher than the theoretically predicted binding energy of the intrinsic exciton in CsPbCl_3_ (approximately 64–72 meV) [42,43]. We think that the sharp increase in *E_a_* is due to the high electronegativity of the Br_2_ molecules incorporated into the crystal lattice, which capture electrons from the lattice. Subsequently, the holes in the crystal approach these electrons, and both are tightly captured nearby the Br_2_ molecules. This strongly binds the electron–hole pair together, and its exciton radius is very small. Due to the small exciton radius, when exposed to external energy, this electron–hole pair will recombine very quickly, exhibiting a very short decay time.

For the low-energy emission peak 2, the thermal activation energy obtained from the Arrhenius fitting is 58.79 eV (Figure 6d), which reflects the decrease in the exciton binding energy after Br is incorporated into the crystal lattice. We think this is because the shallow energy level defects generated after Br is incorporated into the crystal lattice bring many new and rapid radiation recombination sites, thereby causing a significant shortening of the decay time. In summary, the Br incorporation into the CsPbCl_3_ has significantly changed the exciton recombination situation. On the one hand, the highly electronegative Br_2_ molecule greatly shortens the exciton radius, thereby significantly reducing the recombination time. On the other hand, the Br incorporated into the crystal lattice brings shallow energy level recombination sites, and the increase in the recombination pathways leads to a decrease in the decay time.

### 3.6. Alpha Particle Excitation

**Figure 7:**
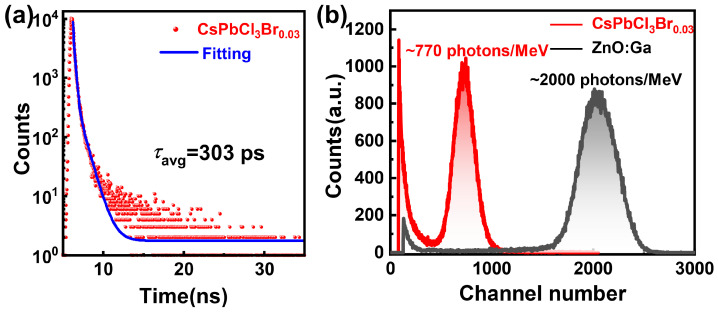
(**a**) Single particle time-resolved spectrum of the CsPbCl_3_Br_0.03_ crystal excited by the 5.486 MeV *α*-particle emitted from ^241^Am. (**b**) Comparison of pulse height spectra (light yield) between the CsPbCl_3_Br_0.03_ crystal and a standard ZnO:Ga scintillator excited by the 5.486 MeV *α*-particle emitted from ^241^Am.

To further verify the fast timing scintillation mechanism of the CsPbCl_3_Br_0.03_ crystal under actual high-energy radiation conditions, we use an ^241^Am *α*-particle source to excite the sample and measured the corresponding decay curve. As shown in Figure 7a, the average decay time of the CsPbCl_3_Br_0.03_ crystal remains in the sub-nanosecond range at 303 ps, which is much shorter than the 652 ps decay time obtained under 375 nm laser excitation. The scintillation rise time is 780 ps, which indicates that the CsPbCl_3_Br_0.03_ single crystal possesses excellent fast timing response. Additionally, using the ^241^Am *α*-particle source as the source, we obtain the pulse height spectrum of the CsPbCl_3_Br_0.03_ crystal, as shown in Figure 7b. Taking the commercial polycrystalline ZnO:Ga scintillator as a reference, the light yield of CsPbCl_3_Br_0.03_ crystal is estimated to be approximately 770 photons per MeV, equivalent to 2541 photons MeV^−1^·ns^−1^, about 76.23% of ZnO:Ga [8]. The relatively low light yield is due to its strong self-absorption effect. As can be seen from Figure S2, there is a large overlap region between the RL emission spectrum and the optical absorption spectrum of the CsPbCl_3_Br_0.03_ single crystal.

### 3.7. X-Ray Imaging

**Figure 8:**
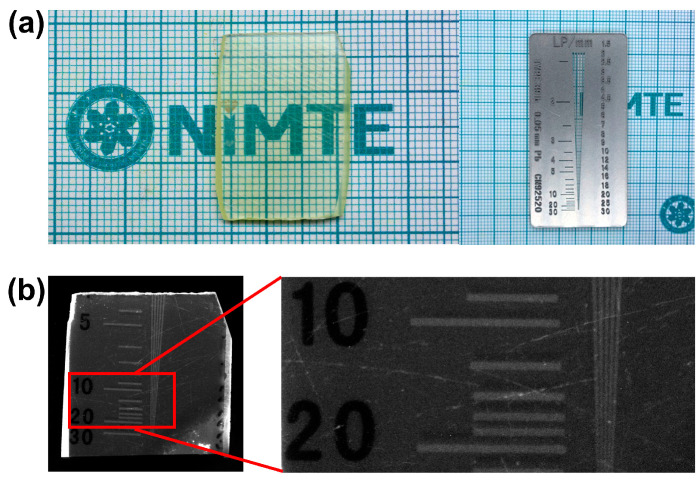
(**a**) Photograph of the CsPbCl_3_Br_0.03_ crystal and the standard TYPE 39b line-pair phantom used for spatial resolution measurements. (**b**) X-ray spatial resolution of the CsPbCl_3_Br_0.03_ crystal measured using the line-pair phantom.

Spatial resolution is a key indicator for evaluating the imaging quality of scintillators under high-energy radiation. We measured the X-ray imaging spatial resolution of the CsPbCl_3_Br_0.03_ crystal using the standard line-pair phantom [44]. The crystal used for imaging and the photo of the line-pair phantom employed are shown in Figure 8a. The radiation source used is a tungsten target X-ray tube with a tube voltage of 50 kV and a tube current of 1000 mA. The line-pair phantom (or imaging target) is placed between the X-ray source and a finely polished 1.48 mm CsPbCl_3_Br_0.03_ crystal. The X-rays emitted from the X-ray source pass through the line-pair card (or imaging target) and are directed onto the crystal, generating visible lights. The visible lights transmit through an internal optical path to a CMOS camera lens with a 20-million-pixel sensor. Figure S3 shows the schematic diagram of the X-ray imaging principle. The exposure time of the camera is set to 15 s, and the X-ray spatial resolution of the CsPbCl_3_Br_0.03_ crystal is measured to be as high as 20 lp/mm, as shown in Figure 8b. This is a significant improvement compared to the best resolution of 4 lp/mm reported in our previous research on similar samples (CsPbCl_3_Br_0.006_, thickness 1.81 mm, exposure time 55 s) [16].

**Figure 9:**
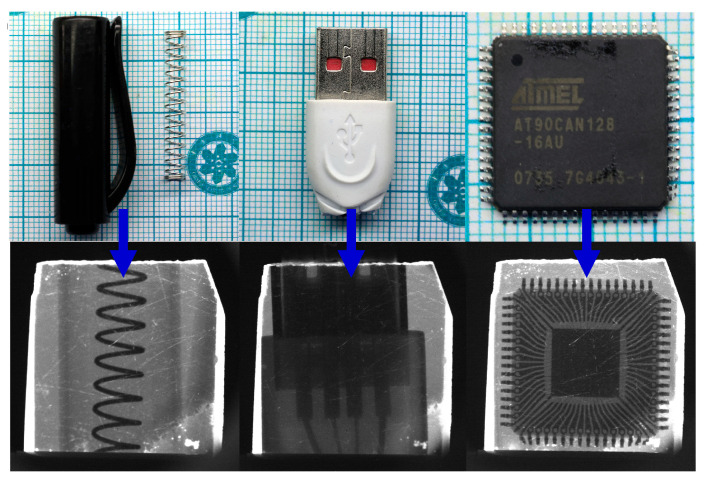
Using the CsPbCl_3_Br_0.03_ crystal for X-ray imaging of different objects, the imaging objects include: a black plastic casing with a metal spring, a USB connector, and a printed circuit board.

In addition, X-ray imaging is also conducted on actual objects, including a black plastic casing with a metal spring, a USB connector, and a printed circuit board, to evaluate the imaging capability of the actual objects, as shown in Figure 9. The X-ray effectively penetrated the non-metallic casing and clearly displayed the image of the metal spring beneath the plastic casing, the internal wiring of the USB connector, and the intricate circuits within the circuit board. We believe that this significant improvement in X-ray spatial resolution is due to the increased crystallinity and excellent optical transparency of the CsPbCl_3_Br_0.03_ crystals grown in this study. The outstanding performance indicates that this material has a possible potential application in X-ray imaging.

## 4. Conclusions

In this study, by meticulously improving the vertical Bridgman growth process of CsPbCl_3_ crystals, we successfully grew a 2-inch-diameter CsPbCl_3_Br_0.03_ single crystal by the vertical Bridgman method. These single crystals are transparent and have high crystalline quality, and compared with pure CsPbCl_3_, the lattice slightly expands. The Br in the crystal lattice introduces high-density fast recombination pathways in the CsPbCl_3_Br_0.03_ crystal and significantly suppresses the slow radioluminescence component. Under *α*-particle excitation, the decay time is shortened to 303 ps, which is significantly shorter than that of pure CsPbCl_3_ and all other perovskites by at least two orders of magnitude. Additionally, the CsPbCl_3_Br_0.03_ wafers function as scintillation screens for X-ray imaging, achieving a spatial resolution of 20 lp/mm under the tested conditions, which can clearly display the fine structure inside the printed circuit board. Through computational calculation, we analyze the possible mechanism for this strategy of growth under Br_2_ overpressure to achieve the fast timing scintillation ability of CsPbCl_3_Br_0.03_. These results indicate that CsPbCl_3_Br_0.03_ has great potential for conceptual fast timing applications, such as time of flight–positron emission tomography. However, due to its obvious internal self-absorption effect, its light yield is relatively low. Future research should focus on identifying the specific Br-related defect species responsible for the fast carrier recombination channel, improving compositional uniformity of the entire ingot, and reducing self-absorption to increase light output while maintaining fast timing scintillation performance.

## Data Availability

The original contributions presented in this study are included in the article/Supplementary Material. Further inquiries can be directed to the corresponding author.

## Supplementary Materials

The following supporting information can be downloaded at: https://www.mdpi.com/article/10.3390/ma19081479/s1, Figure S1: The photo of the pure-phase and colorless CsPbCl_3_ crystal grown in this research; Figure S2: Stokes shift of CsPbCl_3_Br_0.03_ crystals. It shows a strong self-absorption effect, which accounts for the low light yield of the crystal; Figure S3: Schematic diagram illustrating the principle of X-ray imaging; Figure S4: The PXRD measured at different positions of the single crystal ingot grown under Br_2_ overpressure; Figure S5. (a) Schematic diagram of the supercell structure of CsPbCl_3_. (b) Schematic diagram of the supercell structure of CsPbCl_3_ with Br incorporated in the form of single atoms. (c) Schematic diagram of the supercell structure of CsPbCl_3_ with Br incorporated in the form of Br_2_ molecules. The incorporated Br atoms are indicated by red symbols; Figure S6. The SEM-EDS measured at different positions of the single crystal ingot grown under Br_2_ overpressure; Figure S7. The dark current-voltage (*I-V*) measured at different positions of the single crystal ingot grown under Br_2_ overpressure; Table S1. The calculation results of the volume expansion rate and formation energy of the CsPbCl_3_Br_0.03_ models. References [29,30,31,32,33] are cited in Supplementary Materials.
