# Supplementary material for: Realizing 303 ps Ultrafast Scintillation Time in 2-Inch CsPbCl3 Single Crystals Grown Under Br2 Overpressure
Source: Materials (Basel). 2026 Apr 8;19(8):1479. doi: 10.3390/ma19081479 (PMC13117060; doi:10.3390/ma19081479)
Supplement: Supplementary file 1 [file materials-19-01479-s001.zip › materials-4179779-supplementary.pdf]

Supplementary Information

Realizing 303 ps Ultrafast Scintillation Time in 2-Inch $CsPbCl_3$ Single Crystals Grown Under Br_2 Overpressure

Jingwei Yang [1,2,3], Fangbao Wang [4], Liang Chen [4], Tao Bo [1,3], Zhifang Chai [1,3] and Wenwen Lin [1,3,*]

1 Zhejiang Key Laboratory of Data-Driven High-Safety Energy Materials and Applications, Ningbo Key Laboratory of Special Energy Materials and Chemistry, Laboratory of Advanced Nuclear Materials, Ningbo Institute of Materials Technology and Engineering (NIMTE), Chinese Academy of Sciences, Ningbo 315201, China

2 University of Chinese Academy of Sciences, Beijing, 100049, China

3 Qianwan Institute of Ningbo Institute of Materials Technology and Engineering (NIMTE), Ningbo 315336, China

4 State Key Laboratory of Intense Pulsed Radiation Simulation and Effect and Radiation Detection Research Center, Northwest Institute of Nuclear Technology, Xi'an 710024, China

* Correspondence: linwenwen@nimte.ac.cn

Experimental section

Materials

Chemicals were used as obtained: cesium chloride (99.999% purity, Aladdin), lead chloride (99.999% purity, Aladdin), cesium bromide liquid (99.999% purity, Aladdin), bromine (99.5% purity, Hushi).

Cesium tribromide powder was synthesized in a fume hood. First, cesium bromide powder (0.02 mol, 4.2562 g) was mixed with an excess of liquid bromine (0.025 mol, 3.9953 g). Then, the mixture was placed in a round-bottomed flask and left to soak in an oil bath at 80 °C for 1 hour to ensure a complete reaction of $CsBr + Br_2 = CsBr_3$. After all the excess liquid bromine evaporated, the remaining orange product at the bottom of the round-bottomed flask was collected.

Crystal growth and methods

(1) First, a quartz tube was immersed in a 2% hydrofluoric acid (HF) solution for 12 hours. Then, the inner wall of the quartz tube was rinsed with deionized water and

absolute ethanol. Finally, it was dried in a 120 °C oven for 24 hours.

(2) The precursor mixture of CsCl (0.8 mol, 134.688 g) and $PbCl_2$ (0.8 mol, 222.488 g) was loaded into a quartz tube with an inner diameter of 21 mm and an outer diameter of 25 mm. The tube was evacuated and sealed under a residual pressure of approximately 4×10^{-3} mbar. The sealed tube was then placed in a rocking furnace and held at 700 °C for 24 hours to ensure complete melting and reaction, followed by slow cooling to room temperature over 24 hours. The synthesized polycrystalline $CsPbCl_3$ was transferred into a cleaned and dried 2-inch-inner-diameter ampoule equipped with a conical tip. To introduce the Br_2 dopant during single crystal growth, a crucible with an inner diameter of 8 mm and an outer diameter of 10 mm containing $CsBr_3$ powder (0.012 mol, 4.4712 g) was suspended in the vapor zone above the raw material. This setup allowed Br_2 vapor to be released upon heating, while liquid CsBr (melting point 636 °C) remained in the crucible. Finally, the ampoule was evacuated and sealed.

(3) The sealed ampoule was loaded into a Bridgman furnace. The three temperature zones were heated to 670 °C, 570 °C, and 500 °C, respectively, at a ramp rate of 2 °C/min, establishing a temperature gradient of 7.6 °C/cm in the crystallization zone. The melt was soaked at a high temperature for 12 h to ensure complete melting and bubble elimination, as well as to facilitate preliminary diffusion equilibrium between the Br_2 vapor and the liquid phase. The crucible containing $CsBr_3$ was suspended about 10 centimeters above the melt. During the initial high-temperature stage, the temperature at the position of the $CsBr_3$ remained at approximately 670-700 °C. The decomposition of $CsBr_3$ released 0.012 mol of Br_2. Considering that the quartz tube had an inner diameter of 50.8 mm and a height of 18 cm, the initial Br_2 pressure inside the tube was estimated to be approximately 2.6-2.7 atm. As the growth proceeded and the ampoule descended, the local temperature and Br_2 pressure also decreased accordingly.

Crystal processing and characterization

The crystal ingot was sliced perpendicular to the growth axis using a diamond wire saw at a cutting rate of 0.5 mm/min, yielding wafers with a thickness of 2.0 mm. Subsequently, the wafers underwent sequential grinding and precision polishing. They

were first ground using silicon carbide (SiC) abrasive papers with grits of 800, 1500, 3000, 5000, and 7000, followed by polishing with 15000- and 60000-mesh diamond powders to achieve a high-quality, smooth surface. After processing, the final thickness of the wafers was approximately 1.0 mm.

Scanning Electron Microscope–Energy Dispersive Spectrometer (SEM-EDS)

The morphology observation, energy dispersive spectroscopy (EDS) analysis, and elemental mapping measurements of the samples were performed using a Zeiss Sigma 300 thermal field emission scanning electron microscope. The accelerating voltage was set to 15 kV. The proportion of bromine in the as-grown crystals was identified and qualitatively assessed via EDS analysis.

Powder X-ray diffraction (PXRD) measurement

Phase analysis of the ground crystal powder was performed using a Bruker D8 ADVANCE X-ray diffractometer. The measurements were conducted with a Cu $K\alpha$ radiation source (λ=1.5478 Å) operated at a tube voltage of 40 kV and tube current of 40 mA. The patterns were recorded over a 2-theta range of 5° to 90° with a step size of 0.02°.

Charge transport testing

The wafer was processed into dimensions of 5.0×5.0 mm^2 with a thickness of approximately 1.0-1.5 mm and used for charge transport measurements. A high-work-function Au electrode was deposited on one side via magnetron sputtering (30 mA, 300 s), while a low-work-function liquid $Ga_{0.75}In_{0.25}$ alloy electrode was applied to the opposite side. This asymmetric electrode configuration aims to form a Schottky junction to effectively suppress dark current. The device was mounted on an insulating quartz substrate equipped with conductive copper tape pads. Copper wires with a diameter of 0.1 mm were used to connect the crystal electrodes to the copper tape, secured with conductive silver paste to ensure good contact. During testing, the device was shielded in a sealed aluminum box to eliminate ambient light and electromagnetic interference. *I-V* characteristics were measured using a Keithley 6517B electrometer.

The measurements of the bandgap

The Hitachi UH-4150 spectrophotometer was used to measure the optical diffuse

reflection in the 200-2500 nm wavelength range to determine the band gap at room temperature. Barium sulfate was selected as the reference. The sample was ground into powder and compacted. The Tauc plot method was used to determine the band gap. A polished wafer with a thickness of 1 mm was selected to test the transmission spectrum on a PerkinElmer Lambda 1050+. The data acquisition wavelength range was 200-800 nm, with a scan speed of 30 nm/min and an interval of 1 nm.

Measurement of photoluminescence spectroscopy

Steady-state and time-resolved photoluminescence (PL) spectra were acquired using an Edinburgh FLS1000 spectrometer equipped with a 375 nm laser diode (5 mW), a time-correlated single-photon counting (TCSPC) module, and a PMT-900 photomultiplier detector. To avoid surface artifacts and lattice deformation induced by polishing, freshly cleaved surfaces of unpolished crystals were selected for testing. The bulk sample was mounted in a holder and excited by the laser at an incidence angle of 45° from approximately 10 cm. The emission slit width was set to 5 nm. PL spectra were collected in the 400–800 nm range with a step size of 1.0 nm and an integration time of 0.5 s. The TRPL decay curves were analyzed via multi-exponential fitting using the built-in FAST module to determine carrier decay times.

Temperature-dependent PL measurements were performed on wafers mounted on a vertical cryostat cooled by liquid nitrogen. Data were collected from 80 to 300 K at 20 K intervals using 375 nm excitation (5 mW), with the spectral range focused between 400 and 500 nm. A 400 nm long-pass filter was inserted in the detection path for all measurements to effectively block scattered laser light.

X-ray imaging characterization

For the X-ray imaging test, the X-rays are generated from OminiFluo960-XrayP-Z of Zolix equipped with a tungsten target X-ray tube, with a maximum power of 50 W, which can be tuned via changing work current and voltage. The pattern used is a TYPE39b line-pair card. It has a thickness of 0.03 mm and a maximum resolution of 30 line-pairs per millimeter. The distance between the line-pair card and the crystal is approximately 5 mm.

Measurement of single photon resolved spectra

The luminescence decay curves of α-particles were measured using the dual channel single photon correlation (DCSPC) method. In this method, a photomultiplier tube (Hamamatsu, R7899) was employed to record a specific scintillation event, while a microchannel plate (Hamamatsu R3809U-52) was utilized to detect a single photon randomly emitted during the scintillation event. The scintillator was mounted on a photomultiplier tube (Hamamatsu, CR173-Q1) to acquire the pulse height spectrum. The amplified signals were analyzed using a multi-channel analyzer (ORTEC ASPEC-927). A PMT (ET Enterprises, 9815B) was coupled to an oscilloscope (Teledyne LeCory, HDO8108A) for the collection of single particle waveforms. During the testing process, the sample was directly positioned on the receiving window of the PMT. The $CsPbCl_3Br_{0.03}$ sample, the PMT, and the isotope source were placed within a light-tight box. Gamma and α-particles were sourced from ^{241}Am isotope sources, respectively. The crystal sample was placed adjacent to the receiving window of the photomultiplier tube. The radioactive source ^{241}Am was fixed on a platform and located in close proximity to the crystal. All of the aforementioned components were housed within a light-tight enclosure to mitigate the influence of ambient light. The high voltage was supplied by a DC high-voltage source. The signals from the photomultiplier tube were recorded using an oscilloscope with a sampling rate of 10 GHz. For each sample and radioactive source combination, over 1000 waveforms were recorded.

In our system, the instrument response function (IRF) is fast enough, and its FWHM is significantly smaller than the scintillation time of the crystal, so its influence on the measured attenuation curve can be ignored. The reported scintillation time is directly obtained from the raw data through a single exponential fitting, without the need for mathematical deconvolution. The scintillation decay time of the crystal in this article has a single exponential behavior. It was extracted by applying a single exponential fitting to the trailing edge of the pulse waveform, starting from the peak. The rise time is defined as the time interval required for the pulse amplitude to increase from 10% to 90% of its maximum peak value. This ultrafast decay and rise time have high repeatability in multiple samples from different parts of the ingot.

Measurement of light yield

The light yield was determined using a pulse height analysis system comprising a photomultiplier tube (PMT, Hamamatsu CR173-Q1), a preamplifier (ORTEC 113), a spectroscopy amplifier (ORTEC 672), and a multi-channel analyzer (ORTEC ASPEC-927MCB). The scintillator $CsPbCl_3Br_{0.03}$ was optically coupled to the PMT, and the radiation source (^{241}Am) was positioned 2 mm from the sample; the entire assembly was housed in a light-tight box. The PMT detected photon pulses generated by single particles and converted them into electrical signals, which were subsequently amplified by the preamplifier and spectroscopy amplifier before being collected and analyzed by the multi-channel analyzer.

Computational method

All density functional theory (DFT) calculations in this work were performed by using the Vienna Ab-Initio Simulation Package (VASP) [1]. The electron–ion interaction was described by the projector augmented wave (PAW) method [2], and the Perdew–Burke–Ernzerhof generalized gradient approximation (PBE-GGA) exchange-correlation functional was employed [3]. All calculations were performed using a plane-wave cutoff energy of 550 eV and Gaussian smearing with a width of σ = 0.10 eV. The $2 \times 2 \times 2$ and $2\sqrt{2} \times 2\sqrt{2} \times 2$ supercell models of $CsPbCl_3$ were used to calculate the introduction of a single Br atom and Br2 molecule, respectively. Brillouin zone integration was carried out using a Monkhorst–Pack k-point mesh of (1 × 1 × 1) [4]. The self-consistent field (SCF) iterations were considered converged when the energy change fell below 1×10^{-5} eV, and atomic positions were fully relaxed until the maximum force on any atom was less than 0.005 eV $Å^{-1}$. The formation energy of interstitial Br atoms (E_f) is calculated according to the following formula:

$$E_f = (E_{crystal+Br} - E_{crystal} - n_{Br}E_{Br})/n_{Br}$$

where $E_{crystal+Br}$ represents the energy of $CsPbCl_3$ after the incorporation of Br_2, $E_{crystal}$ indicates the energy of $CsPbCl_3$, E_{Br} represents the energy per atom for a Br_2 molecule, and n_{Br} represents the number of Br atoms incorporated into the crystal [5].

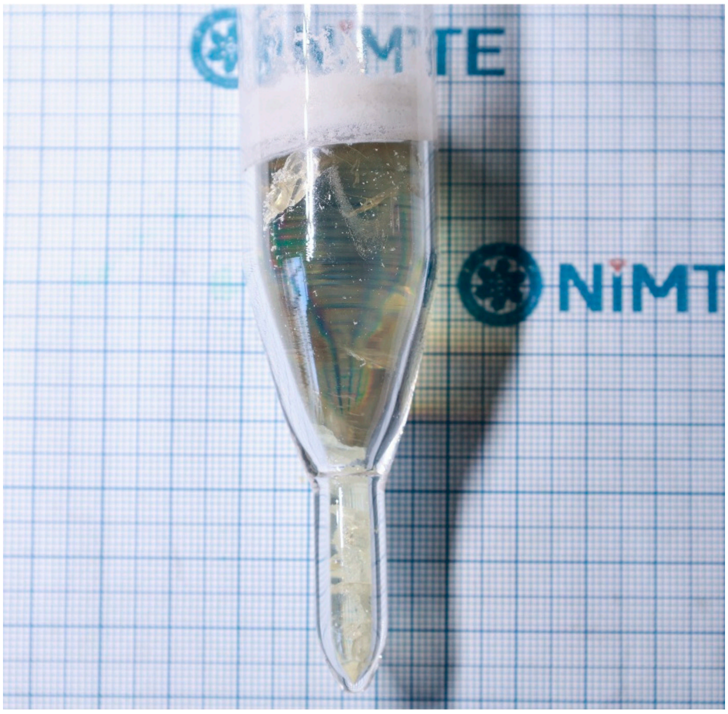

Figure S1: The photo of the pure phase and colorless $CsPbCl_3$ crystal grown in this research.

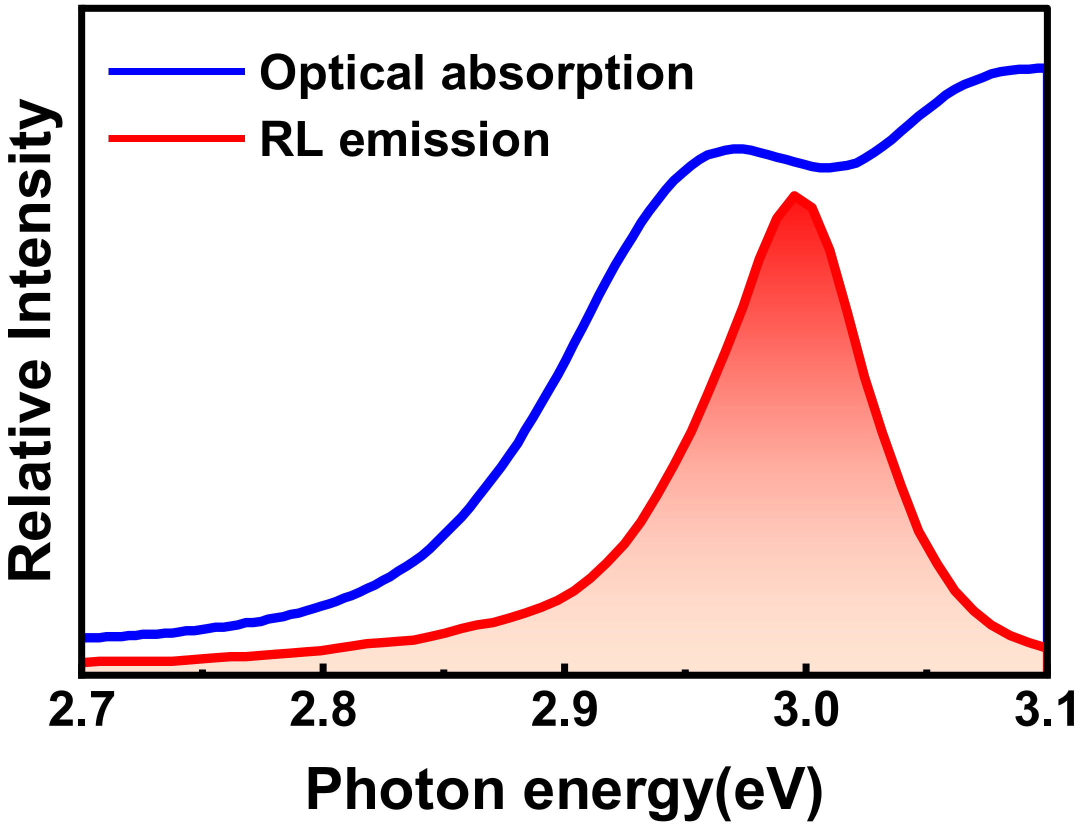

Figure S2: Stokes shift of $CsPbCl_3Br_{0.03}$ crystals. It shows a strong self-absorption effect, which accounts for the low light yield of the crystal.

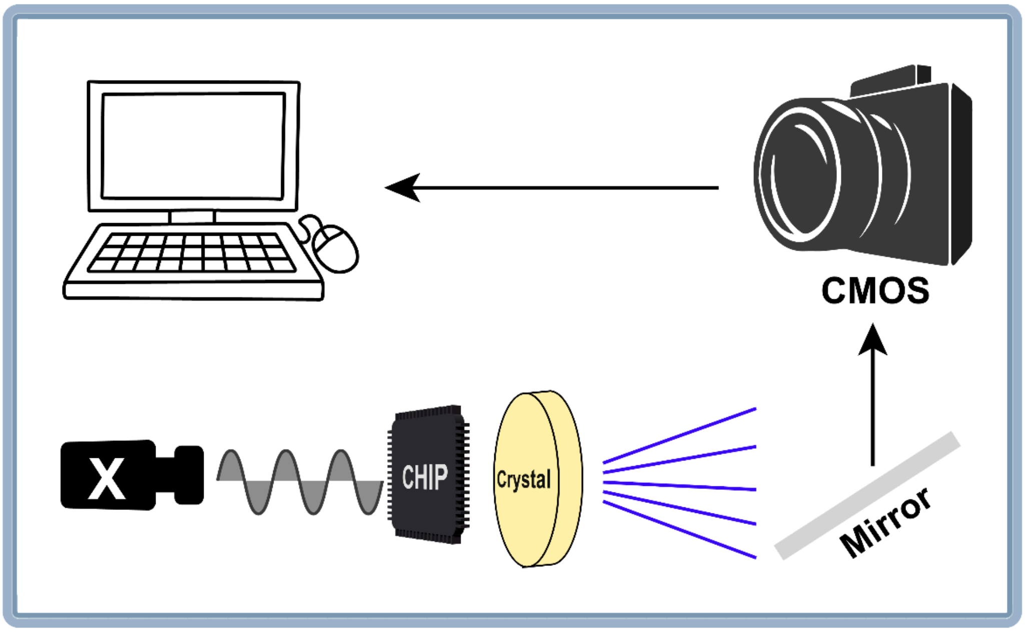

Figure S3: Schematic diagram illustrating the principle of X-ray imaging.

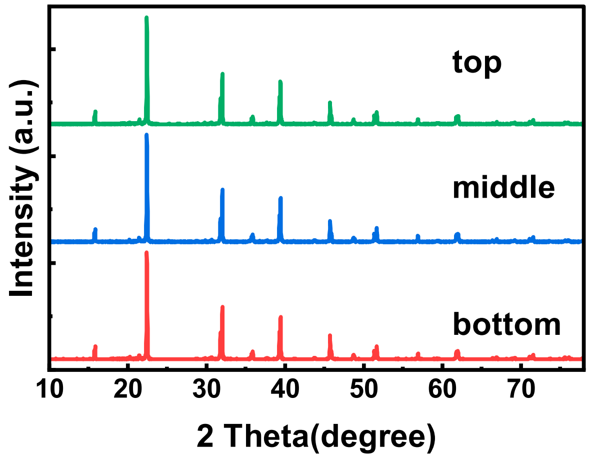

Figure S4: The PXRD measured at different positions of the single crystal ingot grown under Br_2 overpressure.

To figure out in what form the Br elements enter the $CsPbCl_3$ crystal lattice, a $CsPbCl_3$ model was constructed based on density functional theory (DFT) calculations. Individual Br atoms and Br_2 molecules were independently introduced at the interstitial site, positioned at the most stable locations, and the corresponding formation energies

at these positions were calculated. The corresponding three-dimensional structures are shown in Figure S5(a) to S5(c).

The results show that both Br and Br_2 can fill the interstitial site between four adjacent octahedra, and there is a spatial interaction with the surrounding Cs ions. The unit cell volume and formation energy are summarized in Table S1. It is noteworthy that, when the interstitial site is occupied by a Br_2 molecule, the calculated formation energy of the model reaches its value of 0.228 eV, which is smaller than the formation energy of 0.322 eV when a single Br atom enters the interstitial site, indicating that this is a more stable configuration in terms of energy. (The formation energy of the pure $CsPbCl_3$ model is set to 0.) In this case, when the Br incorporated into the crystal lattice exists in the form of Br_2 molecules, the calculated volume expansion rate is approximately 0.59%.

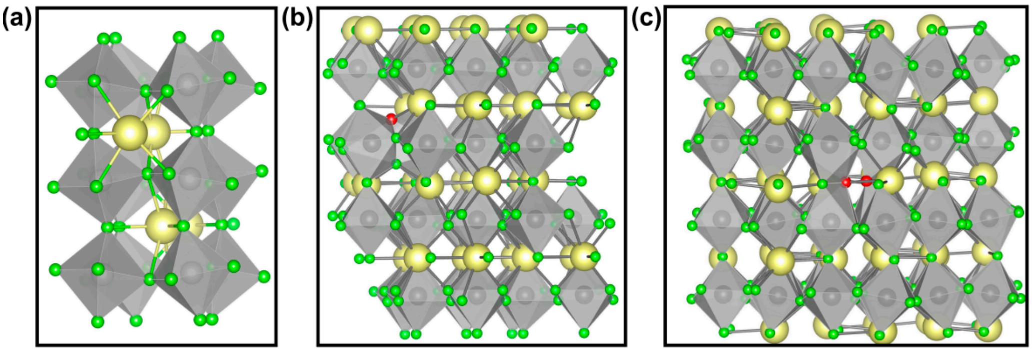

Figure S5. (a) Schematic diagram of the supercell structure of $CsPbCl_3$. (b) Schematic diagram of the supercell structure of $CsPbCl_3$ with Br incorporated in the form of single atoms. (c) Schematic diagram of the supercell structure of $CsPbCl_3$ with Br incorporated in the form of Br_2 molecules. The incorporated Br atoms are indicated by red symbols.

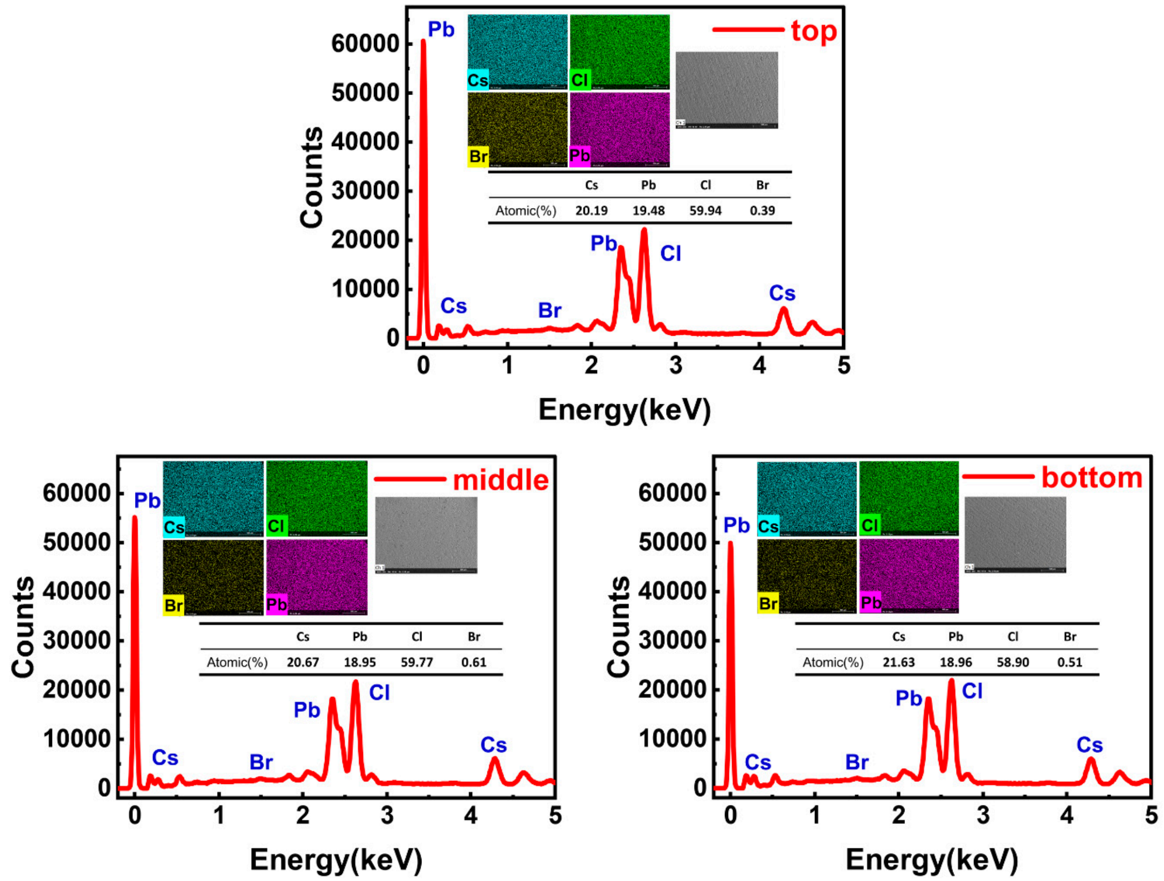

Figure S6. The SEM-EDS measured at different positions of the single crystal ingot grown under Br_2 overpressure.

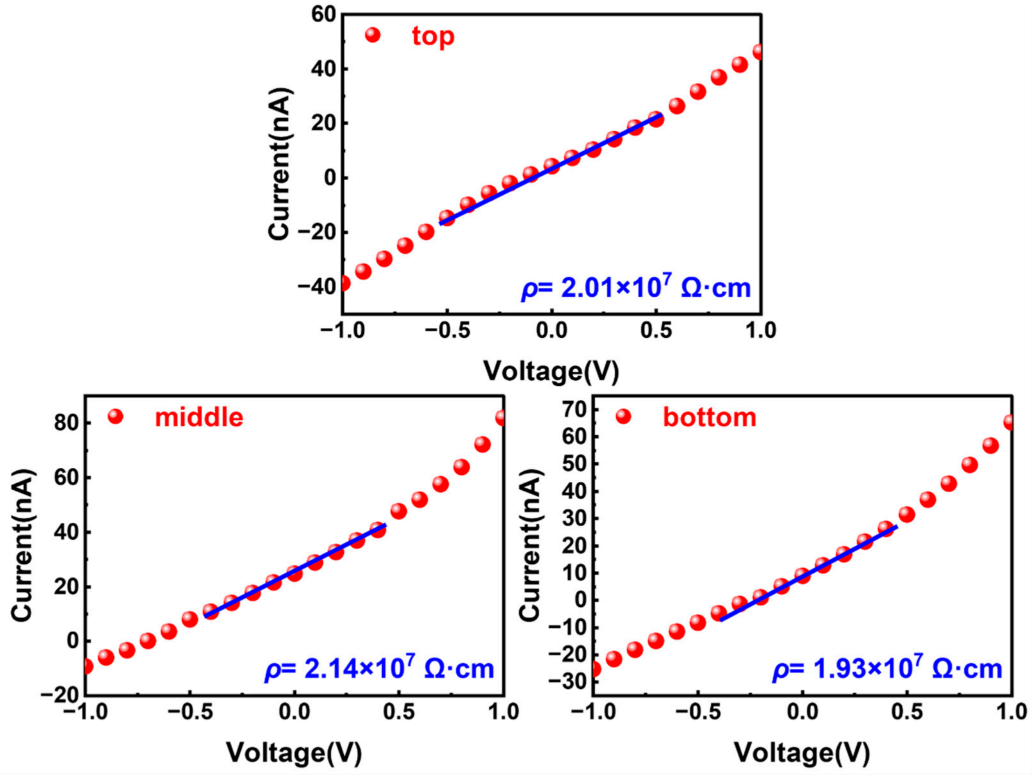

Figure S7. The dark current–voltage (*I-V*) measured at different positions of the single crystal ingot grown under Br_2 overpressure.

Table S1. The calculation results of the volume expansion rate and formation energy of the $CsPbCl_3Br_{0.03}$ models.

The form of Br interstitial site	Expansion rate (%)	Forming energy of Br atom (eV)
Incorporated with a single Br	0.10792	0.322
**Incorporated with Br_2 molecule**	**0.58768**	**0.228**

References:

1. Kresse, G.; Furthmüller, J. Efficient Iterative Schemes for *Ab Initio* Total-Energy Calculations Using a Plane-Wave Basis Set. *Phys. Rev. B* **1996**, *54* (16), 11169–11186. https://doi.org/10.1103/PhysRevB.54.11169.
2. Blöchl, P. E. Projector Augmented-Wave Method. *Phys. Rev. B* **1994**, *50* (24), 17953–17979. https://doi.org/10.1103/PhysRevB.50.17953.
3. Perdew, J. P.; Burke, K.; Ernzerhof, M. Generalized Gradient Approximation Made Simple. *Phys. Rev. Lett.* **1996**, *77* (18), 3865–3868. https://doi.org/10.1103/PhysRevLett.77.3865.
4. Chadi, D. J. Special Points for Brillouin-Zone Integrations. *Phys. Rev. B* **1977**, *16* (4), 1746–1747. https://doi.org/10.1103/PhysRevB.16.1746.
5. Pela, R.R.; Hsiao, C.-L.; Hultman, L.; Birch, J.; Gueorguiev, G.K. Electronic and Optical Properties of Core–Shell InAlN Nanorods: A Comparative Study via LDA, LDA-1/2, mBJ, HSE06, G0W0 and BSE Methods. Phys. Chem. Chem. Phys. 2024, 26, 7504–7514, doi:10.1039/D3CP05295H.
